# RE: Long-Term Outcomes of Sentinel Lymph Node Biopsy for Ductal Carcinoma in Situ

**DOI:** 10.1093/jncics/pkaa079

**Published:** 2020-09-03

**Authors:** Claudia J C Meurs, Marian B E Menke-Pluijmers, Sabine Siesling, Pieter J Westenend

**Affiliations:** 1 CMAnalyzing, Zevenaar, the Netherlands; 2 Department of Surgery, Albert Schweitzer Hospital, Dordrecht, the Netherlands; 3 Department of Research and Development, Netherlands Comprehensive Cancer Centre Organisation, Utrecht, the Netherlands; 4 Department of Health Technology and Services Research, Technical Medical Centre, University of Twente, Enschede, the Netherlands; 5 Laboratory of Pathology Dordrecht, Dordrecht, the Netherlands

We read with interest the article by Hung et al. “Long-Term Outcomes of Sentinel Lymph Node Biopsy for Ductal Carcinoma in Situ” (1). The authors compared patients aged 67-94 years with ductal carcinoma in situ (DCIS) of the breast with a sentinel lymph node biopsy (SLNB) with those without a SLNB and did not find statistically significant differences for treated recurrence, ipsilateral invasive occurrence, and breast cancer mortality. From this, they concluded that the routine performance of SLNB is not warranted in older patients.

We do not think that this conclusion can be drawn based on the data they had available and selected for their analysis. As stated by Hung et al. (2), they used data from patients with a final diagnosis DCIS. These data do not reflect the complete diagnostic work-up. The use of SLNB is not intended as a staging procedure based on a final diagnosis of DCIS in the resection specimen. SLNB is considered a staging procedure, guidance of therapy, and follow-up preoperatively in patients with biopsy-proven DCIS. Of these biopsy-proven patients, 20%-25% will have invasive cancer as a final diagnosis, and this cancer needs to be staged. Staging of these invasive cancers by SLNB is not possible after a mastectomy and possibly less reliable after breast-conserving surgery. In addition, it is more convenient to perform a 1-stage procedure. Performing SLNB in all patients with biopsy-proven DCIS would result in unnecessary procedures in 70%-75% of the patients who will have a final diagnosis of DCIS. Therefore, guidelines have been proposed to identify patients with an increased risk of a final diagnosis of invasive cancer (3, 4). In the article by Huang et al. (2), patients with DCIS and SLNB were of younger age, higher grade DCIS, and larger size and lacked hormone receptor expression compared with patients with DCIS and no SLNB. All of these have been identified as risk factors for occult invasive cancers. The difference in distribution in the study by Huang et al. (2) of these factors in patients with DCIS and SLNB vs without SLNB indicates that the SLNB was done in selected patients, probably according to a guideline.

Guidelines on the use of SLNB in patients with biopsy-proven DCIS are divergent and so is the interpretation of these guidelines, because there also is considerable variation in the actual use of SLNB in these patients (5,6). To improve on this situation, a critical evaluation of the guidelines is necessary but also the development and validation of tools like (web-based) prediction models (nomograms) to support the decision-making process (7). We therefore think it is possible to reduce both the variation and the rate of unnecessary procedures. And thus we think that it is too early to abandon the use of SLNB in patients with biopsy-proven DCIS altogether.

## Note

**Disclosures:** The authors have no disclosures to declare.

## Data Availability Statement

Not applicable.
